# Supplementary material for: Tandem Duplication Events in the Expansion of the Small Heat Shock Protein Gene Family in Solanum lycopersicum (cv. Heinz 1706)
Source: G3 (Bethesda). 2016 Aug 26;6(10):3027–34. doi: 10.1534/g3.116.032045 (PMC5068928; doi:10.1534/g3.116.032045)
Supplement: Supplemental Material [file supp_6_10_3027__index.html]

Tandem Duplication Events in the Expansion of the Small Heat Shock Protein Gene Family in Solanum lycopersicum (cv. Heinz 1706) — Supplemental Material 

# Tandem Duplication Events in the Expansion of the Small Heat Shock Protein Gene Family in *Solanum lycopersicum* (cv. Heinz 1706)

## Supplemental Material for Krsticevic *et al.*, 2016

**Files in this Data Supplement:**

- Figure S1 - Evolutionary relationships between amino acid sequences of 58 putative sHSP genes in *S. lycopersicum* (cv. Heinz 1706) and 11 sHSP orthologous in *A. thaliana*. (.pdf, 199 KB)
- Figure S2 - Putative sHSP genes differentially expressed during fruit development (20 out of a total of 58). (.pdf, 307 KB)
- Figure S3 - The Ka/Ks values for each node in the phylogenetic tree of the sHSP members of Cluster #14. (.pdf, 97 KB)
- Figure S4 - The Ka/Ks values for each node in the gene phylogenetic tree of Cluster#2 sHSP members. (.pdf, 104 KB)
- Table S1 - RNA-Seq datasets used to measure differential expression. (.pdf, 19 KB)
- Table S2 - Transcript abundance of sHSP genes during fruit development and ripening. (.pdf, 135 KB)
- Table S3 - 33 sHSP genes in the *Solanum lycopersicum* (cv. Heinz 1706) genome. (.pdf, 194 KB)
- Table S4 - sHSP gene family in *Solanum lycopersicum* and subcellular localization. (.pdf, 197 KB)
- Table S5 - Top 10 responsive sHSP genes to fruit ripening and heat shock (HS) stress treatments. (.pdf, 177 KB)
