# Supplementary material for: Tandem Duplication Events in the Expansion of the Small Heat Shock Protein Gene Family in Solanum lycopersicum (cv. Heinz 1706)
Source: G3 (Bethesda). 2016 Aug 26;6(10):3027–34. doi: 10.1534/g3.116.032045 (PMC5068928; doi:10.1534/g3.116.032045)
Supplement: Supplemental Material [file supp_g3.116.032045_FigureS1.pdf]

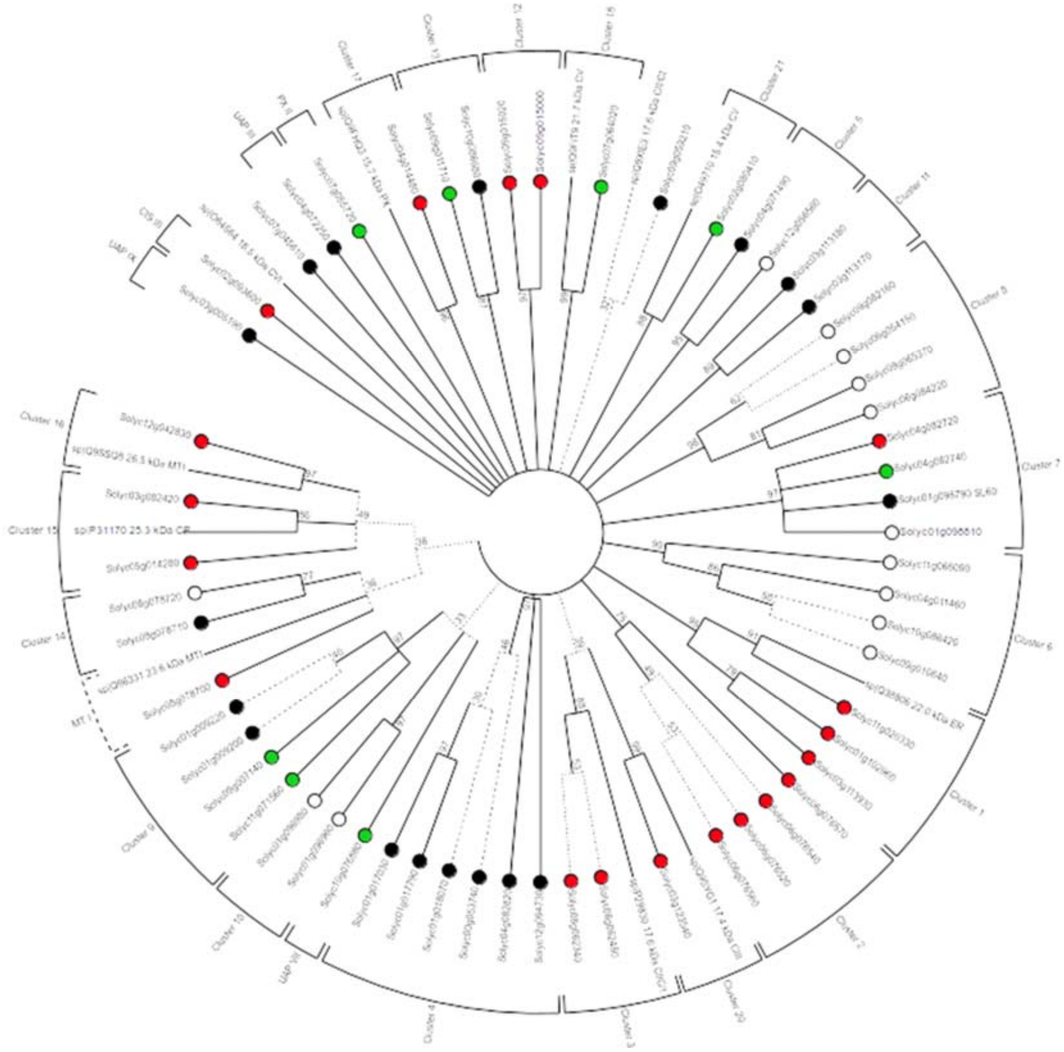

Figure S1. Evolutionary relationships between amino acid sequences of 58 putative sHSP genes in *S. lycopersicum* (cv. Heinz 1706) and 11 sHSP orthologous in *A. thaliana*. The radial tree is enriched with the expression profile of putative sHSP genes in tomato during fruit ripening (MR stage relative to MG reference one). Red and green circle indicate up and down-regulated putative sHSP genes, respectively. White and black circles indicate not differentially expressed (NDE) and not-express (NE) putative sHSP genes, respectively. Clusters with bootstrap values above *70%* are regarded as well supported. Dashed lines indicated branches with bootstrap support below *70%*. Radial tree is drawn condensed, with no branch length proportional to the number of substitutions per site. Clusters were named according literature (Bondino et al., 2012) whenever possible.
