# Supplementary material for: Tandem Duplication Events in the Expansion of the Small Heat Shock Protein Gene Family in Solanum lycopersicum (cv. Heinz 1706)
Source: G3 (Bethesda). 2016 Aug 26;6(10):3027–34. doi: 10.1534/g3.116.032045 (PMC5068928; doi:10.1534/g3.116.032045)
Supplement: Supplemental Material [file supp_g3.116.032045_FigureS2.pdf]

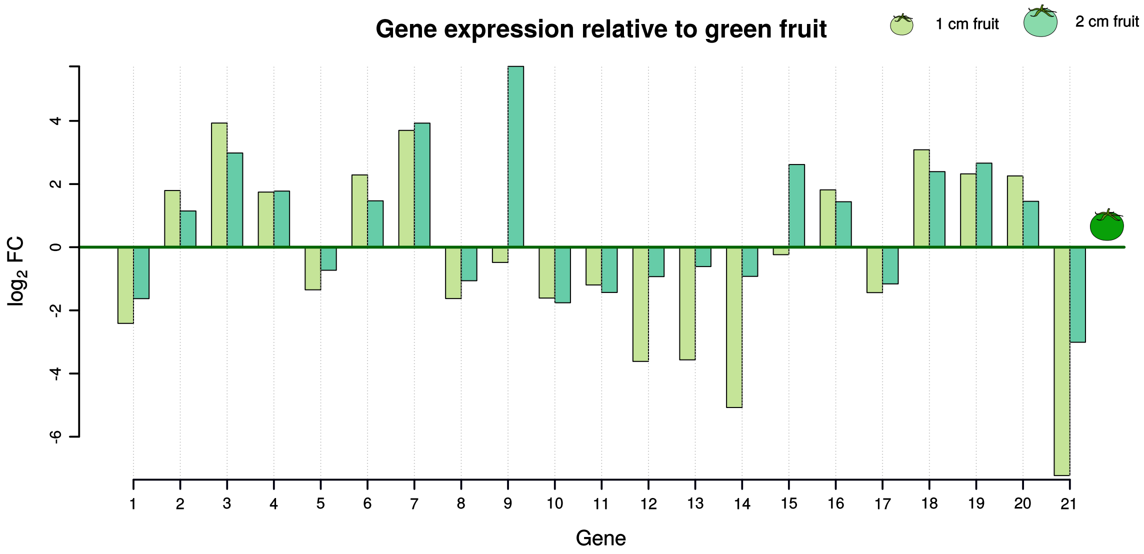

1 - Solyc11g020040
2 - Solyc01g009200
3 - Solyc01g009220
4 - Solyc01g096960
5 - Solyc01g096980
6 - Solyc03g005190
7 - Solyc03g113180
8 - Solyc04g014480
9 - Solyc05g014280
10 - Solyc06g076520
11 - Solyc06g084220
12 - Solyc07g055720
13 - Solyc08g062340
14 - Solyc08g062450
15 - Solyc08g078700
16 - Solyc09g007140
17 - Solyc09g011710
18 - Solyc10g076880
19 - Solyc10g086680
20 - Solyc11g071560
21 - Solyc12g094730

Figure S2. Putative sHSP genes differentially expressed during fruit development (20 out of a total of 58). Differential expression at the 1cm and 2 cm fruit relative to the reference mature green (MG) one is quantified as logFC (base 2). The *hsp70* gene or Solyc11g020040 (#1) is used as a positive control of up-regulation.
