# Supplementary material for: Tandem Duplication Events in the Expansion of the Small Heat Shock Protein Gene Family in Solanum lycopersicum (cv. Heinz 1706)
Source: G3 (Bethesda). 2016 Aug 26;6(10):3027–34. doi: 10.1534/g3.116.032045 (PMC5068928; doi:10.1534/g3.116.032045)
Supplement: Supplemental Material [file supp_g3.116.032045_FigureS3.pdf]

Figure S3 and associated Table. The Ka/Ks values for each node in the phylogenetic tree of the sHSP members of Cluster #14.

Online tools were used to obtain nonsynonymous (Ka) and synonymous (Ks) nucleotide substitution rates (http://services.cbu.uib.no/tools/kaks/). The Ka/Ks ratio should be 1 for genes subject to neutral selection, <1 for genes subject to negative selection, and >1 for genes subject to positive selection. Thus, the Ka/Ks ratio is an indicator of selective pressures on genes, and can be used to identify branches of gene phylogenetic trees were the function of encoded proteins may have changed. The nodes of the tree (1 and 2) are shown in green. Ka and Ks values are shown in blue above each branch. Abbreviations: SL08_720 stands for Solyc08g078720; SL08_710 for Solyc08g078710 and SL08_700 for Solyc08g078700.

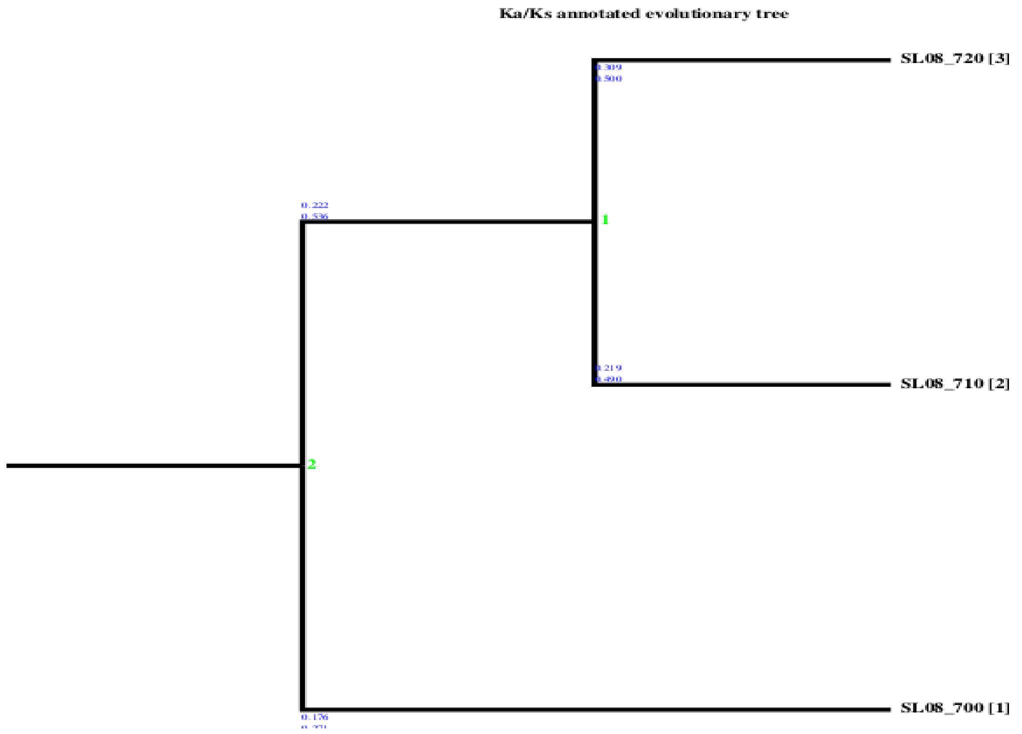

Node #	Ka/Ks Branch1	Ka Branch1	Ks Branch1	Ka/Ks Branch2	Ka Branch2	Ks Branch2
1	1.4227	0.5909	0.4153	1.8975	0.7327	0.3861
2	0.3070	0.0752	0.2451	0.9997	0.5951	0.5952

Associated Table. **DNAsp tool software was used for the computation of occurrence time of the tandem duplicated genes in the Cluster #14 subfamily.** Briefly, DNAsp provides the mean number (Ks) of synonymous substitutions per synonymous site between pairs of duplicated genes. The occurrence time (T) of duplication events can be calculated as T = Ks/2λ, where λ is the clockwise substitution per characteristic of the target species; based on Yang et al., 2008, a value of $\lambda = 1.5 \times 10^{-8}$ corresponding to *A. thaliana* was used.

Seq1	Seq2	SynDif	SynPos	Ks	NSynDif	NSynPos	Ka	Time (T) = Ks/2λ
SL08_700	SL08_710	83,67	112,33	3,7296	188,33	397,67	0,7487	124.320.000,00
SL08_700	SL08_720	85,5	113,67	n.a.	201,5	396,33	0,8496	
SL08_710	SL08_720	79,75	115,17	1,9259	186,25	394,83	0,7436	64.196.666,67
