# Supplementary material for: Tandem Duplication Events in the Expansion of the Small Heat Shock Protein Gene Family in Solanum lycopersicum (cv. Heinz 1706)
Source: G3 (Bethesda). 2016 Aug 26;6(10):3027–34. doi: 10.1534/g3.116.032045 (PMC5068928; doi:10.1534/g3.116.032045)
Supplement: Supplemental Material [file supp_g3.116.032045_FigureS4.pdf]

Figure S4 and associated Table. The Ka/Ks values for each node in the gene phylogenetic tree of Cluster#2 sHSP members.

The Ka/Ks values for each node in the gene phylogenetic tree of Cluster#2 sHSP members. Online tools wereused to obtain nonsynonymous (Ka) and synonymous (Ks) nucleotide substitution rate (http://services.cbu.uib.no/tools/kaks/). The ratio of Ka/Ks nucleotide substitution rates is an indicator of selective pressures on genes, and can be used to identify branches of gene phylogenetic trees, were encoded proteins may have changed function. The Ka/Ks ratio should be 1 for genes subject to neutral selection, <1 for genes subject to negative selection, and >1 for genes subject to positive selection. In green are indicated the nodes of the tree (1, 2 and 3). Ka and Ks values are indicated above each branch in blue. Abbreviations: for SL6_570: Solyc06g076570; SL6_560: Solyc06g076560; SL6_540: Solyc06g076540 and SL6_520: Solyc06g076520.

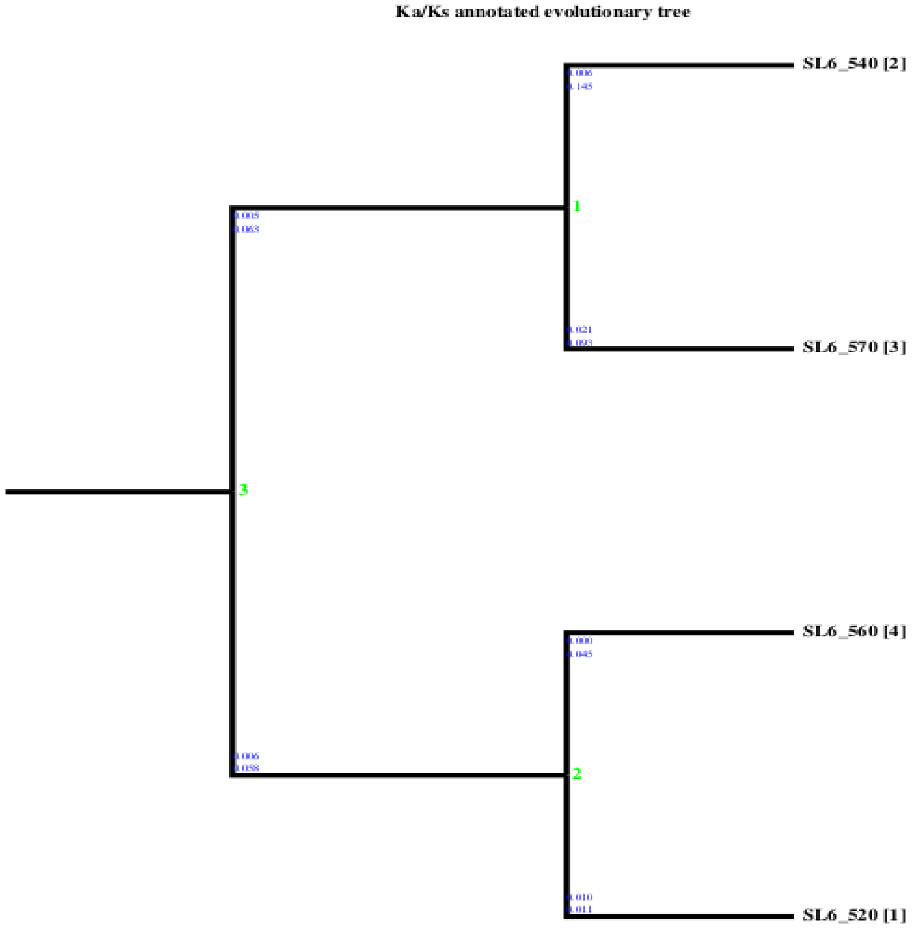

#Node	Ka/Ks Branch1	Ka Branch1	Ks Branch1	Ka/Ks Branch2	Ka Branch2	Ks Branch2
1	0.0410	0.0059	0.1453	0.2277	0.0212	0.0932
2	0	0	0.0446	0.9689	0.0101	0.0105
3	0.0847	0.0053	0.0633	0.1066	0.0062	0.0583

Associated Table. DNAsp tool was used for the computation of occurrence time of the tandem duplicated genes in the Cluster#2 subfamily. Briefly, DNAsp provides the mean number (Ks) of synonymous substitutions per synonymous site between pair of duplicated genes. The occurrence time (T) of duplication events can be calculated using the equation T = Ks/2λ, where λ is the clockwise substitution per characteristic of the target specie; based on Yang et al., 2008, the λ for *A. thaliana* was used.

Gene 1	Gene 2	Ks	T (Myr)
SL6_570	SL6_540	0,3393	11.310.000,00
SL6_540	SL6_520	0,4071	13.570.000,00
SL6_520	SL6_560	0,0648	2.160.000,00
SL6_520	SL6_570	0,2322	7.740.000
