# Supplementary material for: Tandem Duplication Events in the Expansion of the Small Heat Shock Protein Gene Family in Solanum lycopersicum (cv. Heinz 1706)
Source: G3 (Bethesda). 2016 Aug 26;6(10):3027–34. doi: 10.1534/g3.116.032045 (PMC5068928; doi:10.1534/g3.116.032045)
Supplement: Supplemental Material [file supp_g3.116.032045_TableS1.pdf]

Table S1

RNA-Seq datasets used to measure differential expression. The columns show the description, accession number (acc. num.), read length, total number of reads (# reads) and total number of base pairs (# bp) of each dataset.

Description	acc. num.	length	# reads	# bp
Leaf rep. 1	SRR404309	48	11220678	538.6M
Leaf rep. 2	SRR404310	48	10969265	526.5M
Root rep. 1	SRR404311	49	10657563	522.2M
Root rep. 2	SRR404312	49	10650553	521.9M
Flower rep. 1	SRR404313	48	10761639	516.6M
Flower rep. 2	SRR404314	48	10433160	500.8M
Flower bud rep. 1	SRR404315	48	12988759	623.5M
Flower bud rep. 2	SRR404316	48	12711669	610.2M
Fruit immature 1cm rep. 1	SRR404317	49	9158268	448.8M
Fruit immature 1cm rep. 2	SRR404318	49	9572645	469.1M
Fruit immature 2 cm fruit rep. 1	SRR404319	49	7864754	385.4M
Fruit immature 2 cm fruit rep. 2	SRR404320	49	9252491	453.4M
Mature green rep. 1	SRR404324	48	8423857	404.3M
Mature green rep. 2	SRR404325	48	10214504	490.3M
Mature breaking rep. 1	SRR404326	48	11534326	553.6M
Mature breaking rep. 2	SRR404327	48	11111053	533.3M
Mature red rep. 1	SRR404328	48	14085509	676.1M
Mature red rep. 2	SRR404329	48	13515035	648.7M
