# Supplementary material for: Tandem Duplication Events in the Expansion of the Small Heat Shock Protein Gene Family in Solanum lycopersicum (cv. Heinz 1706)
Source: G3 (Bethesda). 2016 Aug 26;6(10):3027–34. doi: 10.1534/g3.116.032045 (PMC5068928; doi:10.1534/g3.116.032045)
Supplement: Supplemental Material [file supp_g3.116.032045_TableS2.pdf]

Table S2. Transcript abundance of sHSP genes during fruit development and ripening. Average FPKM in 1 cm and 2 cm fruit developmental stages and in the Mature Green (MG), Mature Breaker (MB) and Mature Red (MR) fruit ripening stages. Percentages of transcripts abundance relative to the total of sHSP RNAm in the MR stage is also show.

Gene	1cm	2cm	MG	MB	MR	MR/Abundance [%]
Solyc06g076520	92,78	84,44	292,23	1651,64	2554,26	21,823
Solyc06g076560	70,58	68,47	132,12	941,84	2006,14	17,140
Solyc06g076570	149,30	105,05	210,48	1130,00	1962,08	16,764
Solyc05g014280	0,99	75,87	1,46	67,78	829,35	7,086
Solyc09g015000	33,25	42,80	64,99	1025,06	826,07	7,058
Solyc08g062450	1,62	30,02	58,16	404,14	761,02	6,502
Solyc03g082420	7,72	5,90	5,51	148,44	447,95	3,827
Solyc08g078700	26,14	191,53	31,94	199,82	417,25	3,565
Solyc08g062340	3,07	24,61	38,12	563,76	333,37	2,848
Solyc01g102960	4,22	11,21	5,83	155,25	273,53	2,337
Solyc04g014480	24,45	36,69	78,55	258,07	216,77	1,852
Solyc09g015020	11,60	17,00	22,28	125,09	204,24	1,745
Solyc06g076540	9,74	9,51	13,60	63,47	191,62	1,637
Solyc03g113930	1,12	0,34	0,31	21,05	171,54	1,466
Solyc11g020330	3,04	3,93	3,42	65,06	157,85	1,349
Solyc04g082720	54,69	47,68	47,38	59,13	115,68	0,988
Solyc09g011710	70,75	87,37	198,21	125,01	63,70	0,544
Solyc12g042830	2,09	2,14	5,30	29,52	57,83	0,494
Solyc03g123540	23,16	20,82	18,61	20,96	57,58	0,492
Solyc08g078720	57,54	49,11	48,50	25,81	38,61	0,330
Solyc02g093600	1,64	1,68	3,02	21,12	8,16	0,070
Solyc01g098810	11,61	8,42	9,97	9,71	6,29	0,054
Solyc07g064020	6,37	6,13	9,26	5,99	2,32	0,020
Solyc04g082740	3,22	2,94	4,90	1,87	0,51	0,004
Solyc10g086680	4,39	5,71	0,88	0,85	0,32	0,003
Solyc01g098790	0,34	0,65	0,30	0,31	0,31	0,003
Solyc08g078710	0,00	0,11	0,32	0,00	0,06	0,001
Solyc01g009200	2,78	1,83	0,85	0,20	0,05	0,000
Solyc01g009220	1,64	0,83	0,10	0,00	0,00	0
Solyc02g080410	3,64	2,70	1,45	0,11	0,00	0
Solyc04g072250	0,00	0,00	0,11	0,10	0,00	0
Solyc09g007140	5,02	3,97	1,44	0,26	0,00	0
Solyc11g071560	5,09	2,97	1,08	0,24	0,00	0
