# Supplementary material for: Tandem Duplication Events in the Expansion of the Small Heat Shock Protein Gene Family in Solanum lycopersicum (cv. Heinz 1706)
Source: G3 (Bethesda). 2016 Aug 26;6(10):3027–34. doi: 10.1534/g3.116.032045 (PMC5068928; doi:10.1534/g3.116.032045)
Supplement: Supplemental Material [file supp_g3.116.032045_TableS3.pdf]

Table S3. 33 sHSP genes in the *Solanum lycopersicum* (cv. Heinz 1706) genome. Conserved ACD domains are characterized by their number of beta sheets, obtained with the Phyre2 tool (http://www.sbg.bio.ic.ac.uk/phyre2). Additionally, gene annotations reported in the literature (Bondino et al., 2012) and in the Solgenomics database are given (annotation ITAG2.40).

Gene	*# of beta sheets*	*Literature annotation*	*Solgenomics annotation*
Solyc01g009200	9	UAPVII	Restricted tev movement2 IPR008978
Solyc01g009220	8	-	Restricted tev movement2 IPR008978
Solyc01g098790	7	UAPX	with IPR HSP20
Solyc01g098810	9	UAPX	with IPR HSP20
Solyc01g102960	9	ER	CIV
Solyc02g080410	8	UAPI	CI
Solyc02g093600	9	CIsIII	CI
Solyc03g082420	9	P	Choloroplastic
Solyc03g113930	9	ER	CIV
Solyc03g123540	9	CIII	CII
Solyc04g014480	9	PXI	CI
Solyc04g072250	9	UAPIII	CI
Solyc04g082720	9	UAPX	-
Solyc04g082740	9	UAPX	-
Solyc05g014280	9	P	unknown
Solyc06g076520	9	CI	CI
Solyc06g076540	9	-	unknown
Solyc06g076560	9	CI	CI
Solyc06g076570	9	CI	CI
Solyc07g064020	8	UAPVI	CI
Solyc08g062340	9	CII	CII
Solyc08g062450	9	CII	CII
Solyc08g078700	9	MTI	chloroplastic//mitochondrial
Solyc08g078710	9	-	-
Solyc08g078720	7	-	-
Solyc09g007140	9	UAPVII	CI
Solyc09g011710	9	-	CI
Solyc09g015000	9	-	CI
Solyc09g015020	9	CI	CI
Solyc10g086680	9	-	unknown
Solyc11g020330	9	ER	CIV
Solyc11g071560	9	UAPVII	Restricted tev movement2 IPR008978
Solyc12g042830	9	MTII	CI
