# Supplementary material for: Tandem Duplication Events in the Expansion of the Small Heat Shock Protein Gene Family in Solanum lycopersicum (cv. Heinz 1706)
Source: G3 (Bethesda). 2016 Aug 26;6(10):3027–34. doi: 10.1534/g3.116.032045 (PMC5068928; doi:10.1534/g3.116.032045)
Supplement: Supplemental Material [file supp_g3.116.032045_TableS4.pdf]

Table. S4 sHSP gene family in *Solanum lycopersicum* and subcellular localization. sHSP gene clusters (Cluster #) obtained in the phylogenetic tree shown in Figure S1. For each sHSP gene, its protein molecular weight [kDa] deduced from its nucleotide sequence and its amino acid sequence size [#aa] are indicated. Additionally, their correspondent sub-cellular localization and major clades are also indicated. Subcellular localization was obtained using data previously reported in the literature (Bondino et al., 2012) whenever possible. Otherwise, it was predicted using the TargetP program (http://www.cbs.dtu.dk/services/TargetP/). Subcellular localizations are: CI - Class I cytosolic; CII - Class II cytosolyc, CIII - Class III cytosolic; ER - endoplasmic reticulum; PX - perixosomal; MT - mitochondrial; CP - chloroplastic; UAPI - *undefined*. The methodology used to define subcellular localization is shown between brackets: PE - confirmed by previous experiments; TgtP - predicted by the TargetP program; PhyP - predicted by the Phylogeny-based annotation.

Cluster #	*Gene*	*Prot. Mol. Weight/Size*	*Subcellular Localization*	*Major Clades*
2	Solyc06g076520<br>Solyc06g076540<br>Solyc06g076560<br>Solyc06g076570	17.735/154<br>17.591/154<br>17.645/154<br>17.632/154	CI (PE; PhyP)<br>CI (PE; PhyP)<br>CI (PE; PhyP)<br>CI (PE; PhyP)	MCI
12	Solyc09g015020<br>Solyc09g015000	17.662/154<br>15.225/ 134	CI (PhyP)<br>CI (PhyP)	
3	Solyc08g062340	17.624/158<br>17.335/155	CII (PE; PhyP)	
20	Solyc03g123540	16.145/144	CIII (PhyP)	
1	Solyc11g020330<br>Solyc01g102960<br>Solyc03g113930	21.45/190<br>21.65/189<br>21.48/188	ER (PE)<br>ER (PE)<br>ER (PE;PhyP)	
17	Solyc04g014480	16.06/145	PX (PE;PhyP)	
21	Solyc02g080410	15.69/ 137	UAPI (PE)	
Unclustered	Solyc02g093600	15.65/136	CI sIII (PE)	
13	Solyc09g011710<br>Solyc10g086680	24.54/208<br>27.1/234	Cytosolic/nuclear(TgtP)<br>MT(TgtP)	
14	Solyc08g078710<br>Solyc08g078720<br>Solyc08g078700	21.54/196<br>18.23/168<br>23.84/210	MTII (PhyP)<br>MTII (PhyP)<br>MTII (PE; PhyP)	MCII
16	Solyc12g042830	8.96/79	MTI (PE; PhyP)	
18	Solyc07g064020	21.57/188	Cytosolic/nuclear (PhyP)	

15	Solyc03g082420	26.23/ 235	CP (PE; PhyP)	
	Solyc05g014280	25.728/221	CP (PE; PhyP)	
9	Solyc01g009200	25.73/232	Cytosolic/nuclear (TgtP)	MCIII
	Solyc01g009220	23.83/ 213	Cytosolic/nuclear (TgtP)	
	Solyc09g007140	26.79/ 236	Cytosolic/nuclear (TgtP)	
	Solyc11g071560	27.55/ 247	Cytosolic/nuclear (TgtP)	
7	Solyc04g082720	16.72/153	Cytosolic/nuclear (TgtP)	MCIV
	Solyc04g082740	21.63/197	Cytosolic/nuclear (TgtP)	
	Solyc01g098790	54.05/ 487	Cytosolic/nuclear (TgtP)	
	Solyc01g098810	24.57/230	Cytosolic/nuclear (TgtP)	
Unclustered	Solyc04g072250	17.93/ 163	CI (PhyP)	-
