# Supplementary material for: Tandem Duplication Events in the Expansion of the Small Heat Shock Protein Gene Family in Solanum lycopersicum (cv. Heinz 1706)
Source: G3 (Bethesda). 2016 Aug 26;6(10):3027–34. doi: 10.1534/g3.116.032045 (PMC5068928; doi:10.1534/g3.116.032045)
Supplement: Supplemental Material [file supp_g3.116.032045_TableS5.pdf]

**Table S5. Top 10 responsive sHSP genes to fruit ripening and heat shock (HS) stress treatments**. sHSP genes are distributed in a ubiquitous fashion through subcellular compartments. Subcellular localizations are: CI - Class I cytosolic; CII - Class II cytosolyc; ER - endoplasmic reticulum; PX - perixosomal; MT – mitochondrial and CP - chloroplastic. Treatments are: fruit ripening, heat shock (HS) stress in leaves and microspores. Ripe fruit expressed sequences are measured in log2 fold change at the MR relative to the reference MG stage.

| Ranking | Ripe fruit[1] | Localization | HS-leaves [2] | Localization | HS-microspores[3] | Localization |
|---|---|---|---|---|---|---|
| 1 | Solyc05g014280 | CP | Solyc02g093600 | CIsIII | Solyc12g042830 | MTII |
| 2 | Solyc03g113930 | ER | Solyc04g014480 | PX | Solyc03g082420 | CP |
| 3 | Solyc03g082420 | CP | Solyc05g014280 | CP | Solyc11g020330 | ER |
| 4 | Solyc01g102960 | ER | Solyc03g113930 | ER | Solyc06g076560 | CI |
| 5 | Solyc11g020330 | ER | Solyc03g082420 | CP | Solyc05g014280 | CP |
| 6 | Solyc06g076560 | CI | Solyc06g076560 | CI | Solyc06g076520 | CI |
| 7 | Solyc06g076540 | CI | Solyc11g020330 | ER | Solyc08g078700 | MTI |
| 8 | Solyc08g078700 | MTI | Solyc12g042830 | MTII | Solyc08g062340 | CII |
| 9 | Solyc08g062450 | CII | Solyc06g076570 | CI | Solyc04g014480 | PXI |
| 10 | Solyc09g015000 | CI | Solyc08g062450 | CII | NA | NA |

[1] Our study; [2] Fragkostefanakis et al., 2015; [3] Frank et al., 2009.
